# Supplementary material for: CASCADE: high-throughput characterization of regulatory complex binding altered by non-coding variants
Source: Cell Genom. 2022 Feb 9;2(2):100098. doi: 10.1016/j.xgen.2022.100098 (PMC8896503; doi:10.1016/j.xgen.2022.100098)
Supplement: Document S1. Table S6 and Figures S1–S7 [file mmc1.pdf]

Cell Genomics, Volume *2*

## Supplemental information

# CASCADE: high-throughput characterization of regulatory complex binding altered by non-coding variants

**David Bray, Heather Hook, Rose Zhao, Jessica L. Keenan, Ashley Penvose, Yemi Osayame, Nima Mohaghegh, Xiaoting Chen, Sreeja Parameswaran, Leah C. Kottyan, Matthew T. Weirauch, and Trevor Siggers**

**Table S6. Antibodies used for experiments, Related to Figures 2, 3, 4, and 5.** The antibodies listed were used for Western Blots, PBM experiments or both.

| Antibody | Catalog Number | Application |
|---|---|---|
| **Primary Antibodies** | | |
| P300 | ab14984 | PBM Experiment/Western Blot |
| SMARCA4 | sc17796 | PBM Experiment/Western Blot |
| GCN5 | sc-365321x | PBM Experiment/Western Blot |
| RBBP5 | a300-109A | PBM Experiment/Western Blot |
| TBLX1R1 | sc-100908 | PBM Experiment |
| HDAC1 | ab7028 | PBM Experiment |
| MED1 | a300-793a | PBM Experiment |
| BRD4 | a3910-985A50 | PBM Experiment |
| P65 | sc-372X | PBM Experiment |
| P65 | sc-8008 | Western Blot |
| IRF8 | sc-6058X | PBM Experiment |
| IRF3 | D83B9 | PBM Experiment |
| IRF2 | sc-374327 | PBM Experiment |
| PU.1 | sc-352X | PBM Experiment |
| **Secondary Antibodies** | | |
| Donkey anti-goat IgG (H+L) Cross-Adsorbed Secondary Antibody, Alexa Fluor 488 | A11055 | PBM Experiment |
| Goat anti-mouse IgG (H+L) Highly Cross-Adsorbed Secondary Antibody, Alexa Fluor 488 | A11029 | PBM Experiment |
| Goat anti-rabbit IgG (H+L) Highly Cross-Adsorbed Secondary Antibody, Alexa Fluor 488 | A11034 | PBM Experiment |
| Goat anti-mouse IgG (H+L) Highly | A32728 | PBM Experiment |

| | | |
|---|---|---|
| Cross-Adsorbed Secondary Antibody, Alexa Fluor 647 | | |
| Goat anti-rabbit IgG (H+L) Highly Cross-Adsorbed Secondary Antibody, Alexa Fluor 647 | A32733 | PBM Experiment |
| HRP conjugated Goat anti-mouse | G-21234 | Western Blot |
| HRP conjugated Goat anti-rabbit | G-21040 | Western Blot |

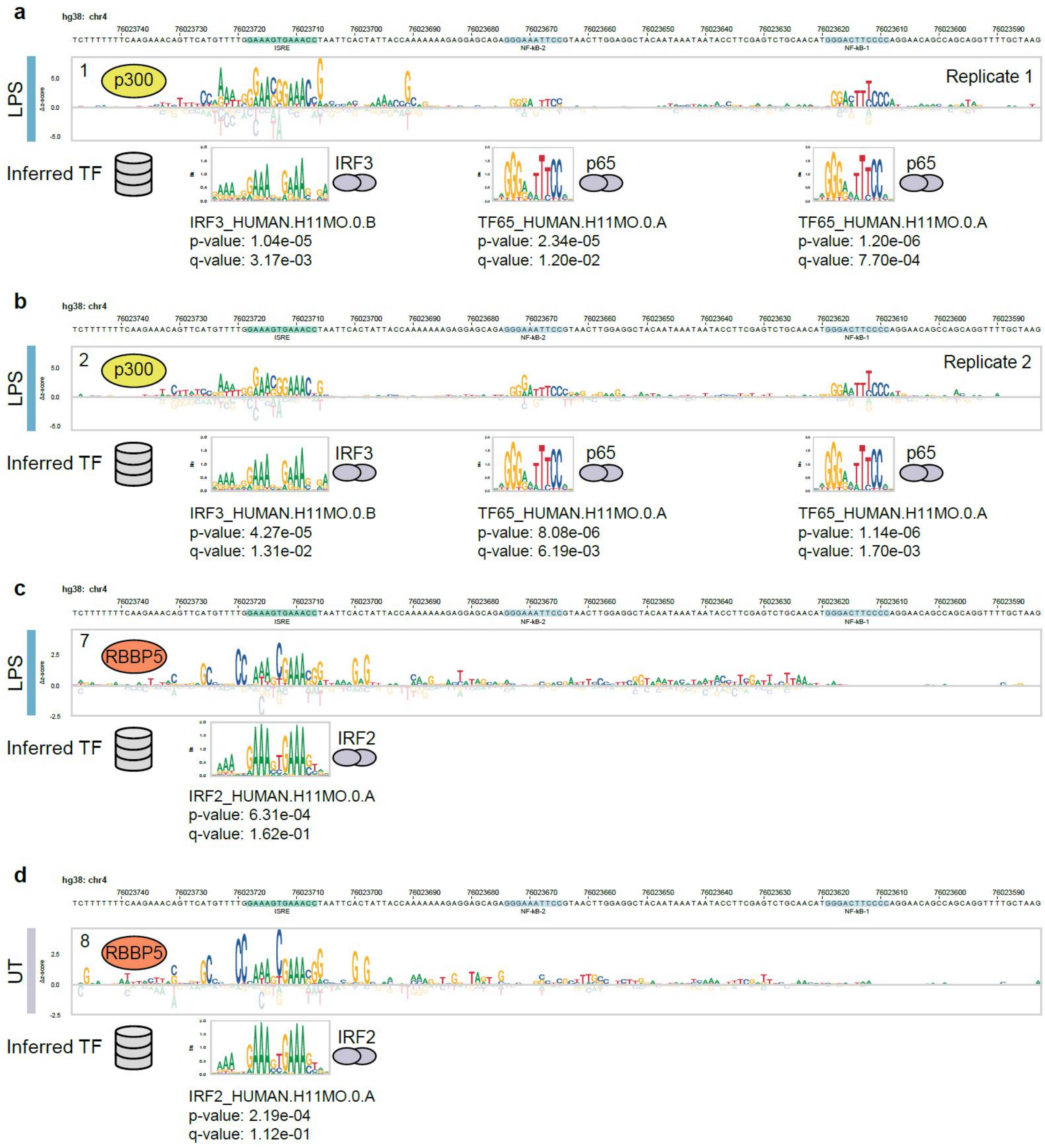


**Figure S1. Model-based inference of transcription factors associated with CXCL10 promoter cofactor recruitment motifs, Related to Figure 2.** (**a**) TF motifs matched to p300 recruitment preferences in LPS-stimulated macrophages (Replicate 1). (**b**) TF motifs matched to p300 recruitment preferences in LPS-stimulated macrophages (Replicate 2). (**c**) TF motif

matched to RBBP5 recruitment preferences in LPS-stimulated macrophages (**d**) TF motif matched to RBBP5 recruitment preferences in untreated macrophages. All COF recruitment preference tracks were converted to probability-based models (see Methods) prior to comparison. Similarity comparisons to known TF binding models was performed using TOMTOM and the full HOCOMOCOv11 motif database (771 total motifs – see Methods). COF: transcriptional cofactor, TF: transcription factor, ISRE: Interferon Stimulated Response Element, NF-kB: Nuclear Factor kappa light chain enhancer of activated B cells, LPS: Lipopolysaccharide, UT: Untreated.

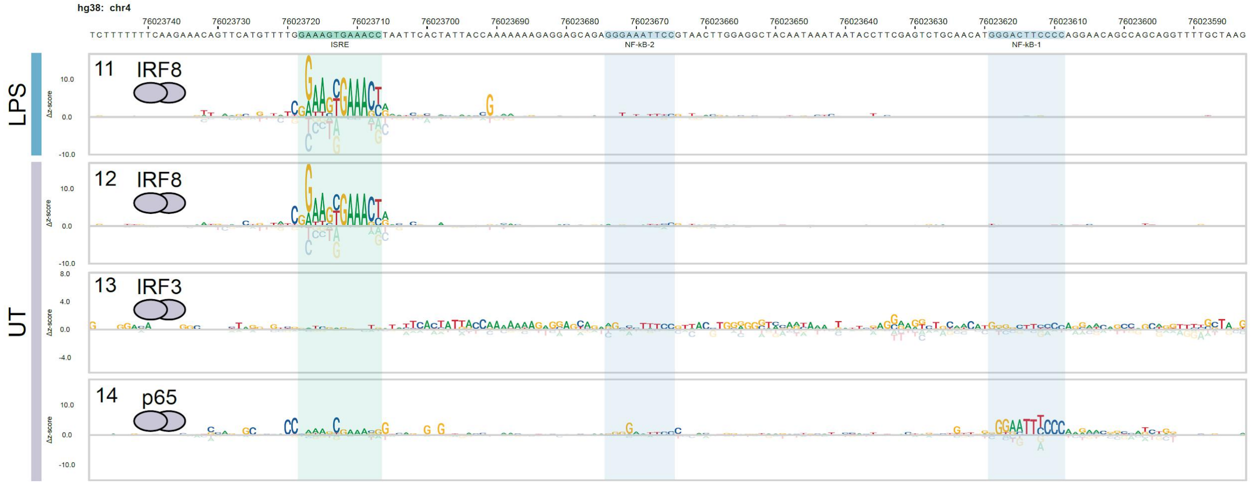


**Figure S2. Additional CASCADE-based analyses of transcription factor binding to the CXCL10 promoter segment, Related to Figure 2.** Nucleotide binding preferences of IRF8 to the CXCL10 promoter segment in paired LPS-stimulated (track 11 - continued from Figure 2) and untreated (track 12) macrophages. Binding preferences of IRF3 (track 13) and p65 (track 14) in untreated macrophages. ISRE: Interferon Stimulated Response Element, NF-kB: Nuclear Factor kappa light chain enhancer of activated B cells, LPS: Lipopolysaccharide, UT: Untreated.

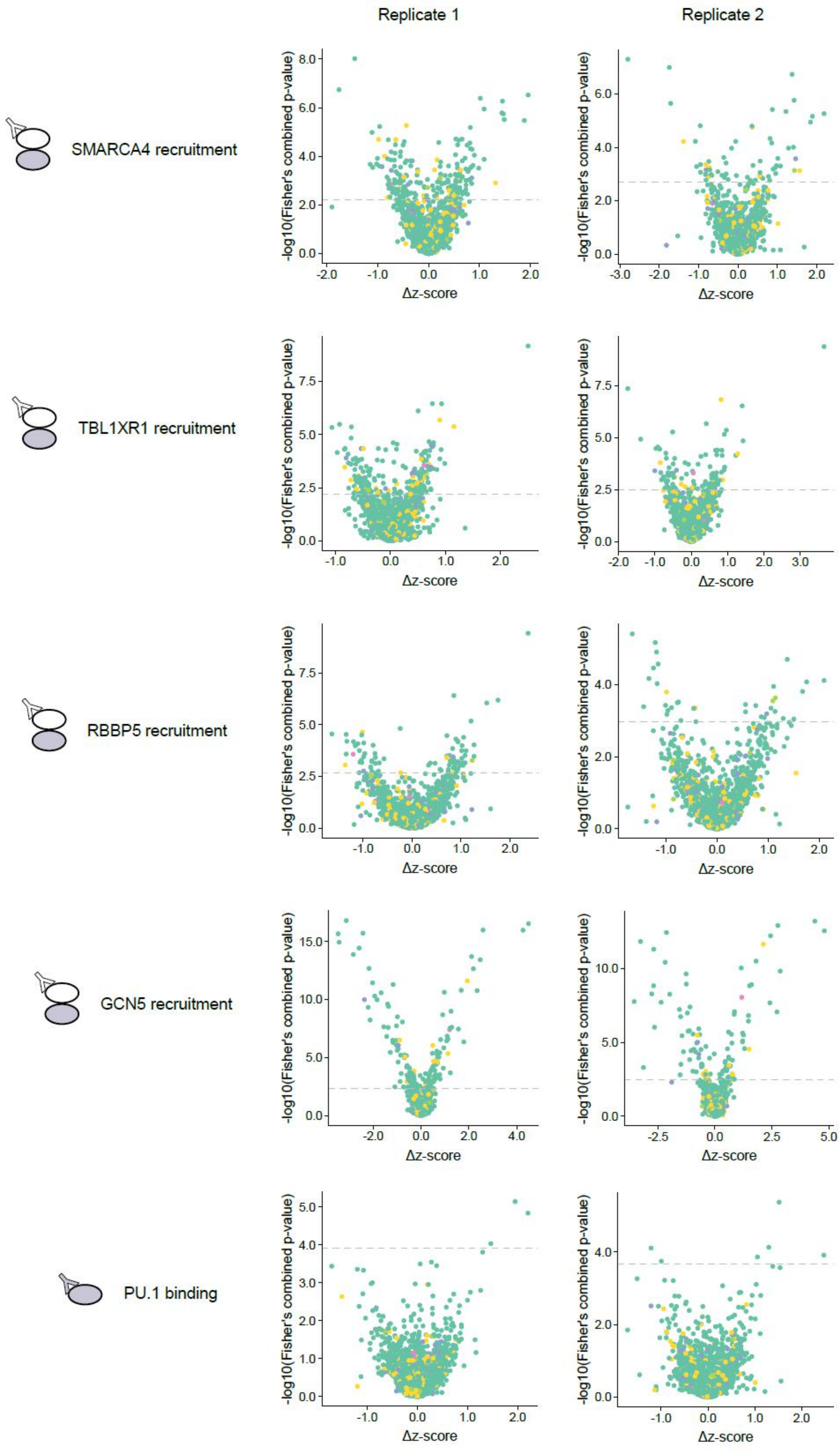

Replicate 1
Replicate 2
SMARCA4 recruitment
TBL1XR1 recruitment
RBBP5 recruitment
GCN5 recruitment
PU.1 binding
-log10(Fisher's combined p-value)
Δz-score

**Figure S3. Statistical significance and direction-of-effect for changes in cofactor recruitment and transcription factor binding across reference and SNP probe pairs screened, Related to Figure 3**. Rows represent volcano plots obtained for different COFs (SMARCA4, TBL1XR1, RBBP5, GCN5) and TF PU.1. Left column shows the volcano plots obtained in a first replicate and the right column shows the volcano plots obtained in a technical replicate experiment. Statistical significance threshold for each experiment ($q < 0.05$, see Methods) is shown as a grey dashed line. COF: transcriptional cofactor, TF: transcription factor.

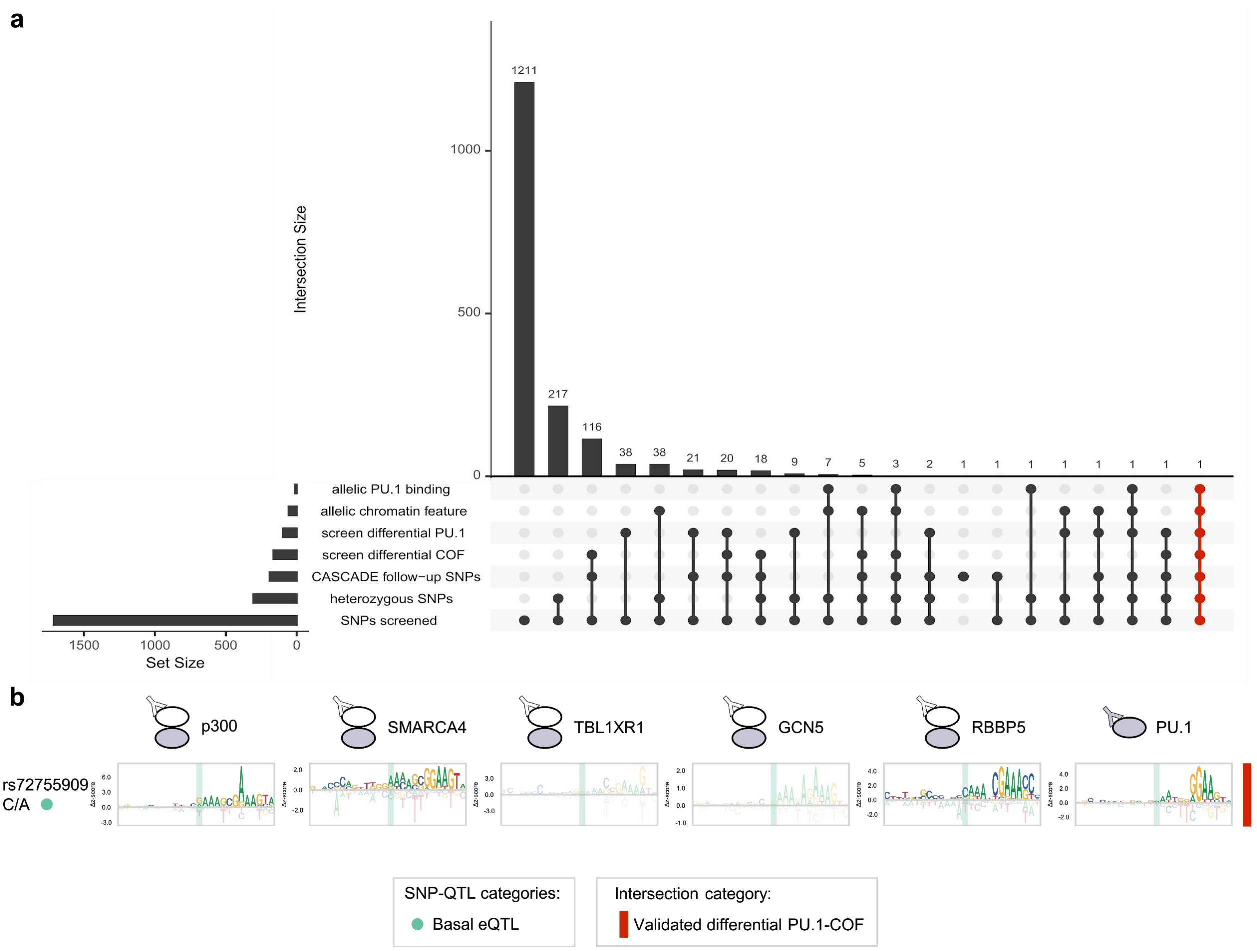


**Figure S4. Comparison of differential cofactor recruitment screen with allelic imbalance data, Related to Figures 3 and 4.** (**a**) UpSet plot demonstrating the size of each set intersection shown. Numbers at the top of each set intersection correspond to the number of SNP-QTLs found within that set intersection. Allelic PU.1 and chromatin feature sets were generated using publicly available ChIP-seq data (see Methods). Differential PU.1 binding and COF recruitment (sets labeled “screen differential PU.1” and “screen differential COF”) were obtained from an array-based COF recruitment and TF binding assay (see Methods). SNPs in the “CASCADE follow-up SNPs” correspond to the set characterized in detail using the CASCADE technique. Heterozygous SNPs in the THP-1 cell line were determined from genotyping array data (see Methods). The set intersection marked in red shows the single example of a SNP that perturbs PU.1 binding in both ChIP-seq and our assay (**b**) COF

recruitment logos for the rs72755909 profiled using CASCADE for p300, SMARCA4, TBL1XR1, GCN5, and RBBP5 recruitment as well as PU.1 binding. The red set intersection indicated is the same as in (**a**).

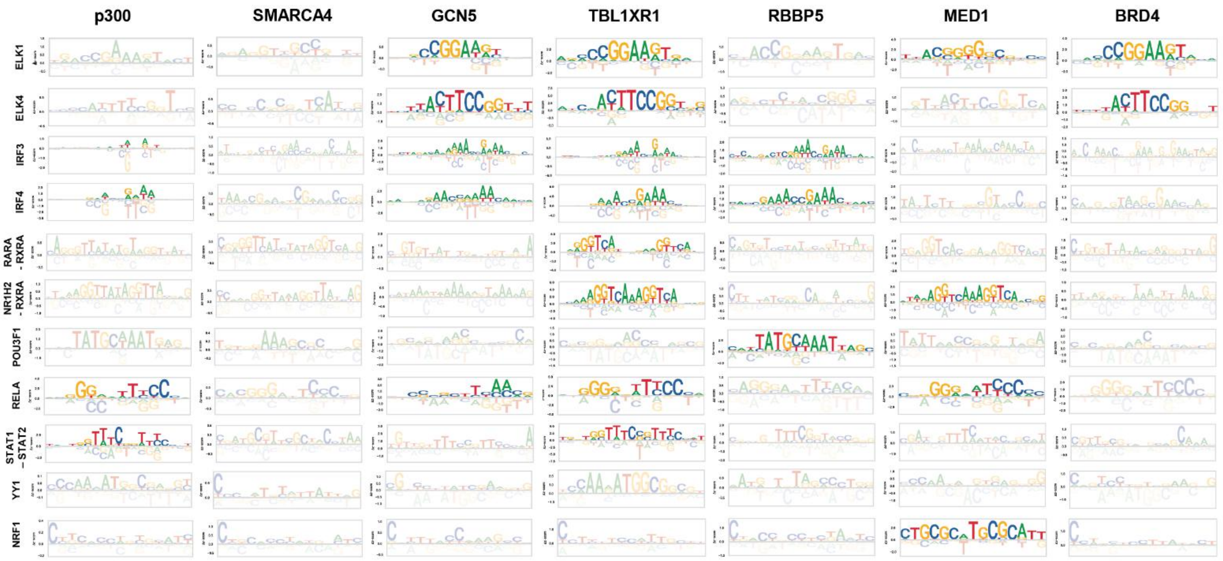


**Figure S5. Profiling cofactor recruitment to a broad set of transcription factor consensus sites, Related to Figures 3, 4, and 5.** COF recruitment motifs were determined by profiling COF recruitment to ~350 transcription factor (TF) consensus sites (and all single-nucleotide variants) (see Methods). COF recruitment motifs for p300, SMARCA4, GCN5, TBL1XR1, RBBP5, MED1, and BRD4 are shown for select TF binding sites to which binding was significant in at least one experiment. Motifs are only included if COF binding to the TF consensus sequence was of sufficient affinity that it met an imposed z-score threshold (z-score > 1.5, see Methods). Motifs shown were found to match the JASPAR motifs for the TFs indicated on the left hand side of each row..

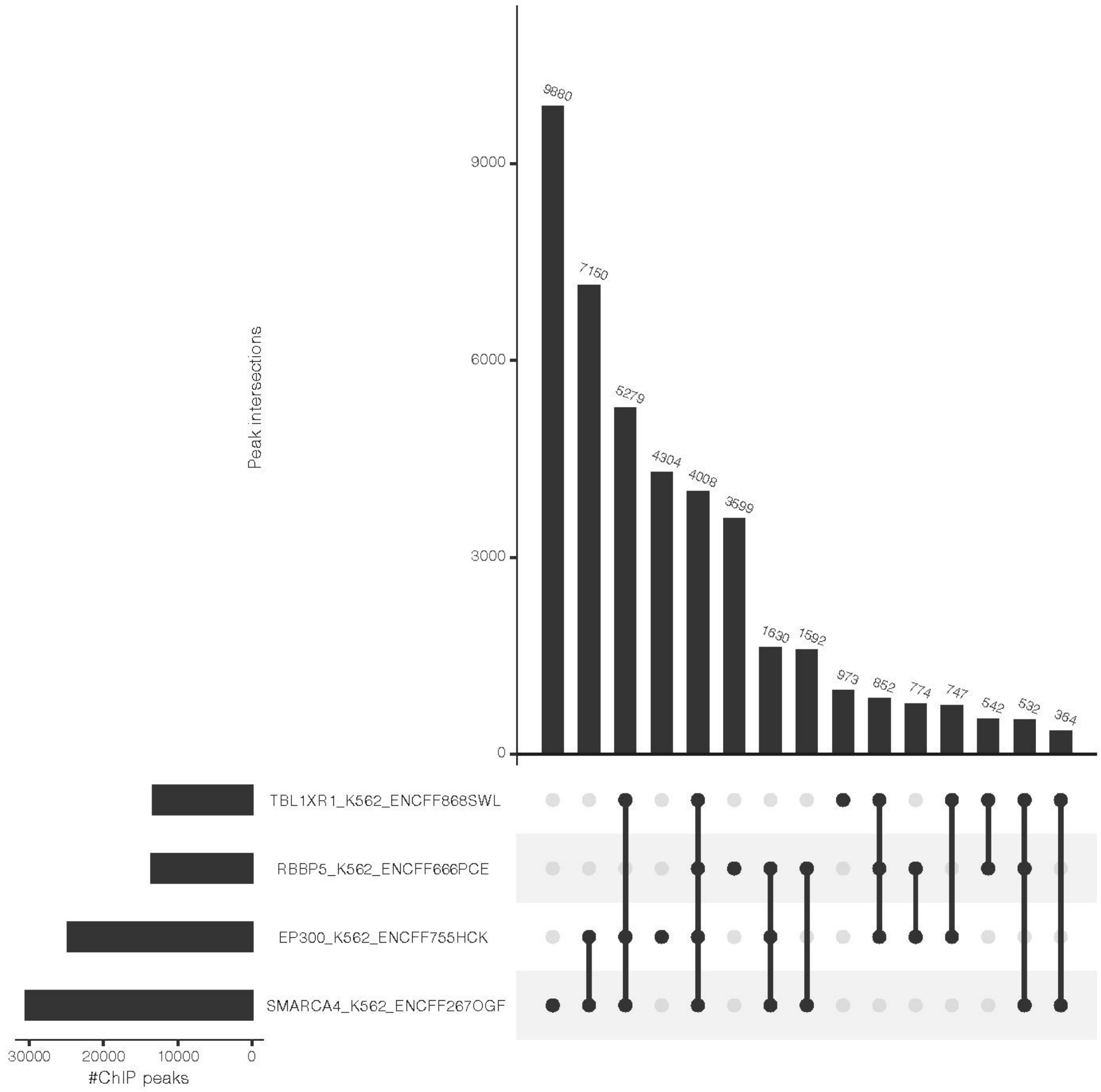


**Figure S6. Overlap of ChIP-seq peaks for four cofactors in K562 cells, Related to Figures 3, 4, and 5.** Shown is an UpSet plot (generated using R) quantifying the level of binding overlap for four of our target COFs in ENCODE ChIP-seq data sets from K562 cells. The ENCODE dataset identifiers are shown for each dataset. Overlap was counted if 1-bp from each ChIP-seq peak overlapped.

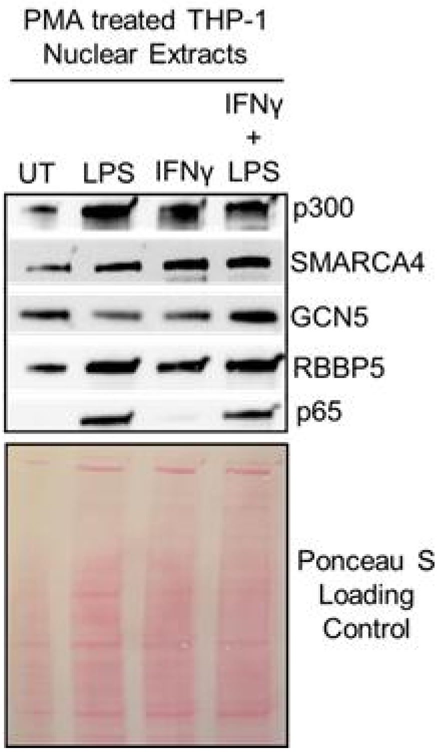


**Figure S7. Western blot of PMA treated THP-1 nuclear extracts, Related to Figures 2, 3, 4, and 5.** The protein expression levels of p300, SMARCA4, GCN5, RBBP5, and p65 of PMA treated THP-1 cells were evaluated by western blotting. 30ug of nuclear extract were loaded for all samples. PMA treated THP-1 cells were treated with LPS for 45 min to induce p65 expression. PMA-treated THP-1 cells were treated with IFNγ for 3 h to prime the immune response. PMA-treated THP-1 cells were treated with IFNγ for 1 h and LPS were treated with IFNγ for 2 h followed by LPS stimulation for 45 min. Ponceau S staining was used as a loading control. UT: untreated, LPS: Lipopolysaccharide, IFNγ: Interferon gamma, IFNγ + LPS: Interferon gamma and lipopolysaccharide.
